# Sidewall angle tuning in focused electron beam-induced processing

**DOI:** 10.3762/bjnano.15.40

**Published:** 2024-04-23

**Authors:** Sangeetha Hari, Willem F van Dorp, Johannes J L Mulders, Piet H F Trompenaars, Pieter Kruit, Cornelis W Hagen

**Affiliations:** 1 Department of Imaging Physics, Delft University of Technology, Lorentzweg 1, 2628CJ Delft, Netherlands; 2 Delmic B.V., Oostsingel 209, 2612 HL Delft, Netherlands; 3 Uniresearch B.V., Delftechpark 37j, 2628 XJ, Delft, Netherlands; 4 Thermo Fisher Scientific, Achtseweg Noord 5, 5651 GG Eindhoven, Netherlands

**Keywords:** electron lithography, FEBID, FEBIE, FEBIP, side wall angle

## Abstract

Structures fabricated using focused electron beam-induced deposition (FEBID) have sloped sidewalls because of the very nature of the deposition process. For applications this is highly undesirable, especially when neighboring structures are interconnected. A new technique combining FEBID and focused electron beam-induced etching (FEBIE) has been developed to fabricate structures with vertical sidewalls. The sidewalls of carbon FEBID structures have been modified by etching with water and it is shown, using transmission electron microscopy imaging, that the sidewall angle can be tuned from outward to inward by controlling the etch position on the sidewall. A surprising under-etching due to the emission of secondary electrons from the deposit was observed, which was not indicated by a simple model based on etching. An analytical model was developed to include continued etching once the deposit has been removed at the exposed pixel. At this stage the secondary electrons from the substrate then cause the adsorbed water molecules to become effective in etching the deposit from below, resulting in under-etched structures. The evolution of the sidewall angle during etching has also been experimentally observed in a scanning electron microscope by continuously monitoring the secondary electron detector signal.

## Introduction

Focused electron beam-induced processing (FEBIP) is a technique in which a focused electron beam is directed onto a substrate with an adsorbed layer of precursor molecules. The precursor molecules are supplied from a gas injection system through a nozzle at close distance to the electron beam focus. The interaction of the incident and scattered electrons with the substrate and adsorbed precursor layer causes the dissociation of the precursor molecules. This results in either deposition of solid precursor fragments (focused electron beam-induced deposition, FEBID) or the removal of substrate material by reactive precursor fragments, that is, etching (focused electron beam-induced etching, FEBIE). For the interested reader, the literature contains a number of good reviews of the technique [1–5].

**Figure 1:**
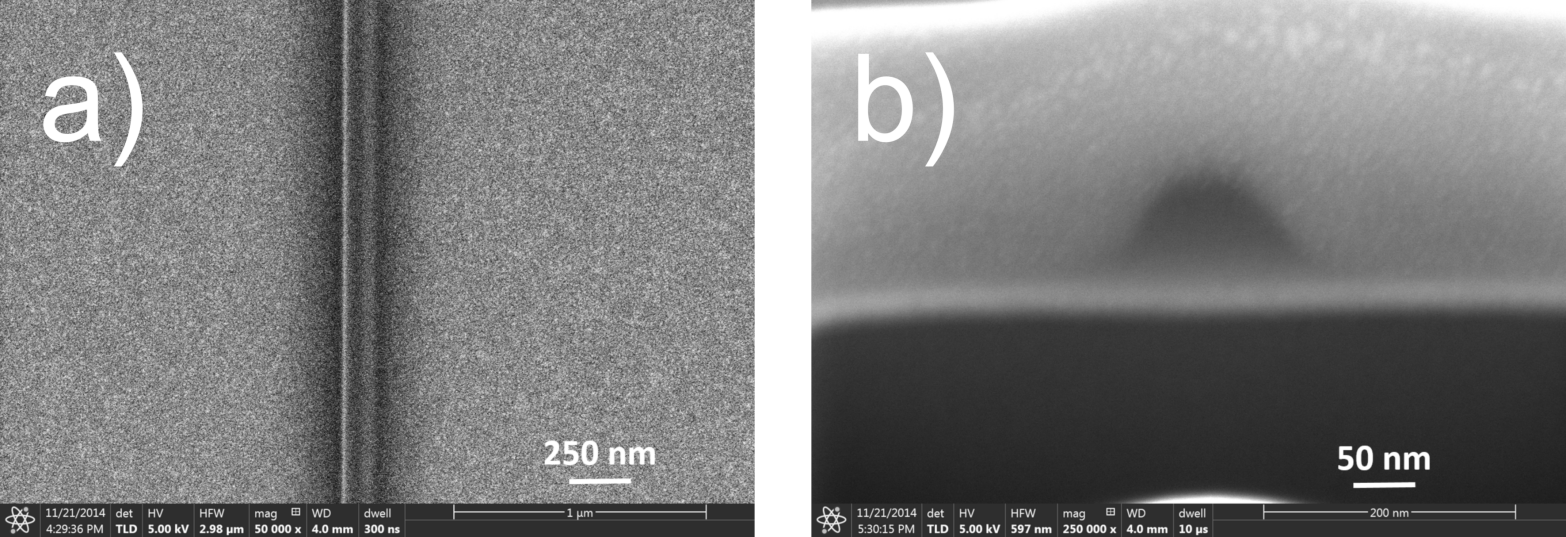
SE image of a deposited FEBID carbon line, top view (a) and FIB cross section (b). The line was deposited from a dodecane precursor on a silicon substrate with a 20 nm gold–palladium layer and a 5 nm titanium adhesion layer. The line was patterned in 500 passes with a dwell time of 500 µs, using a 5 keV beam and 100 pA current with a defocus of 100 nm. Prior to the FIB milling the line was covered with a protective layer of FEBID Pt/C from the MeCpMe_3_Pt precursor.

The cross section of a line patterned using FEBID typically has a Gaussian shape with long tails. This is caused by a combination of the Gaussian current distribution in the primary electron (PE) beam and the spatial distribution of scattered electrons, consisting of backscattered electrons (BSE) and secondary electrons originating from the PE beam (SE1) and from the BSE (SE2) [6–8]. An example of a line deposited from a carbon precursor on a silicon substrate, coated with a 20 nm Au–Pd layer and a 5 nm Ti adhesion layer, is shown in Figure 1a, clearly showing the broad (black) tails on both sides of the line. The cross section of the line, made using focused ion beam (FIB) milling and shown as an electron tilt image in Figure 1b, clearly demonstrates the Gaussian shape. For lithography applications, however, both the long tails and the Gaussian cross section are highly undesirable. The tails may form interconnects to neighboring lines, and the Gaussian cross section will lead to pattern infidelity in subsequent pattern transfer into the underlying substrate.

The aim of this work is to use FEBIE to modify the sidewalls of as-deposited FEBID lines in order to obtain vertical sidewalls. The paper is organised as follows. First, the idea is explained, based on a simple model of the physics involved. Then experimental results are presented, which are quite surprising and call for an extension of the simple model. A more advanced model simulating the FEBIE-assisted sidewall modification is proposed. The simulation results are shown to be in good qualitative agreement with the experimental observations. As a demonstration, the proposed method is applied to a carbon FEBID structure whose sidewall is etched using FEBIE with water in an SEM, using SE signal monitoring to determine when a vertical sidewall has been achieved.

## Results

### Sidewall slope modification – proof of principle simulation

Low-energy electrons are assumed to be most effective in the dissociation process. The reason is that low-energy electrons interact more efficiently with molecules than high-energy electrons. One dissociation channel is dissociative electron attachment (DEA), which occurs when the electron energy matches that of an anion state. Other dissociation channels, such as neutral dissociation (ND) and dissociative ionization (DI), are threshold processes, but their efficiency declines above roughly 100 eV because the interaction time with the molecule becomes too short.

**Figure 2:**
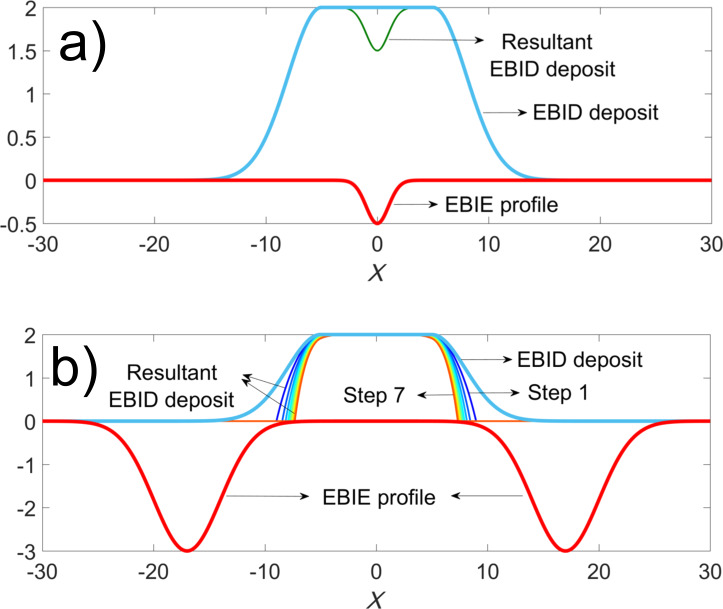
Simulated etching by FEBIE of a planar FEBID surface (a) and the evolution of the sloped sidewalls in seven consecutive etching steps (b). The etching strength in (a) was chosen to be smaller than in (b). With each etching step the sidewalls become steeper, indicated by the seven coloured curves going from blue to orange.

The SE1 are distributed close to the primary beam, while the low-density SE2 are spread out over a much larger area. For simplicity, the spatial distribution of low-energy electrons around the point of impact of the primary beam with the substrate is assumed to be of a Gaussian shape. Depending on the precursor used, a point exposure then results in either a Gaussian shaped deposit (FEBID) or in a Gaussian shaped pit (FEBIE), assuming that the deposition/etching process is proportional to the number of available electrons. In addition, it is assumed that the substrate will not be etched by the FEBIE process. In Figure 2a, a cross section of a simulated FEBID line is shown with a flat top in the middle and sidewalls described by two half Gaussian functions. It is noted here that a FEBID line is deposited as an array of partly overlapping point exposures, which leads to a structure with a flat top and Gaussian sidewalls. Also shown, as a negative Gaussian function, is the etching profile resulting from a point exposure. Note that this is just a two-dimensional model, that is, it represents the cross section of the line and it has zero length in the perpendicular direction.

When FEBIE is applied to the flat top of the line, the etch pit shown as the green curve in Figure 2a results. But when the etching occurs on the sloped sidewall, the secondary electron yield is assumed to increase by 1/cos α(*x*), where α(*x*) is the angle between the incident beam and the normal to the surface at the point of incidence *x* [9]; thus, the etching is enhanced by the same factor. Directing the beam to a fixed position on the sloped sidewall, the Gaussian profile, multiplied by the local SE-yield enhancement factor, governs the etching of the deposit, given a fixed etching strength. Figure 2b illustrates the evolution of the sidewall etching in seven consecutive etching steps, the location of etching being fixed as indicated by the red etching profile. The sidewalls clearly move inwards, approaching the vertical, indicated by the seven coloured curves going from blue to orange (see Supporting Information File 1, section S1 for more details on the simulation).

### Sidewall slope evolution under FEBIE

To experimentally study the modification of the sidewalls of a FEBID structure, carbon structures were first deposited on a silicon substrate with a 20 nm gold–palladium layer and a 5 nm titanium adhesion layer. The coating ensures a strong SE contrast with the carbon deposit due to its significantly higher SE yield, and it prevents the eventual electron beam-induced decomposition of the native oxide layer of the Si substrate. For the carbon deposition, dodecane was used as a precursor. For FEBIE, water was chosen as the etchant, using crystals of MgSO_4_·7H_2_O as a precursor. The experimental section contains more detailed information on the experimental setup and the choice of parameters. It is well known that water acts as an etchant of carbon under electron exposure [10]; it has recently been used to purify FEBID structures by removing the undesired carbon remnants from organometallic precursors [5,11–18]. Another example is a study of the etching rate and the etching profiles achieved in FEBIE with water on diamond samples [19]. Most studies were limited to etching of planar surfaces. Although etching on a slope has been experimentally demonstrated with the slimming of nanowires [20], the shape evolution during etching on sloped surfaces has not been studied thoroughly.

**Figure 3:**
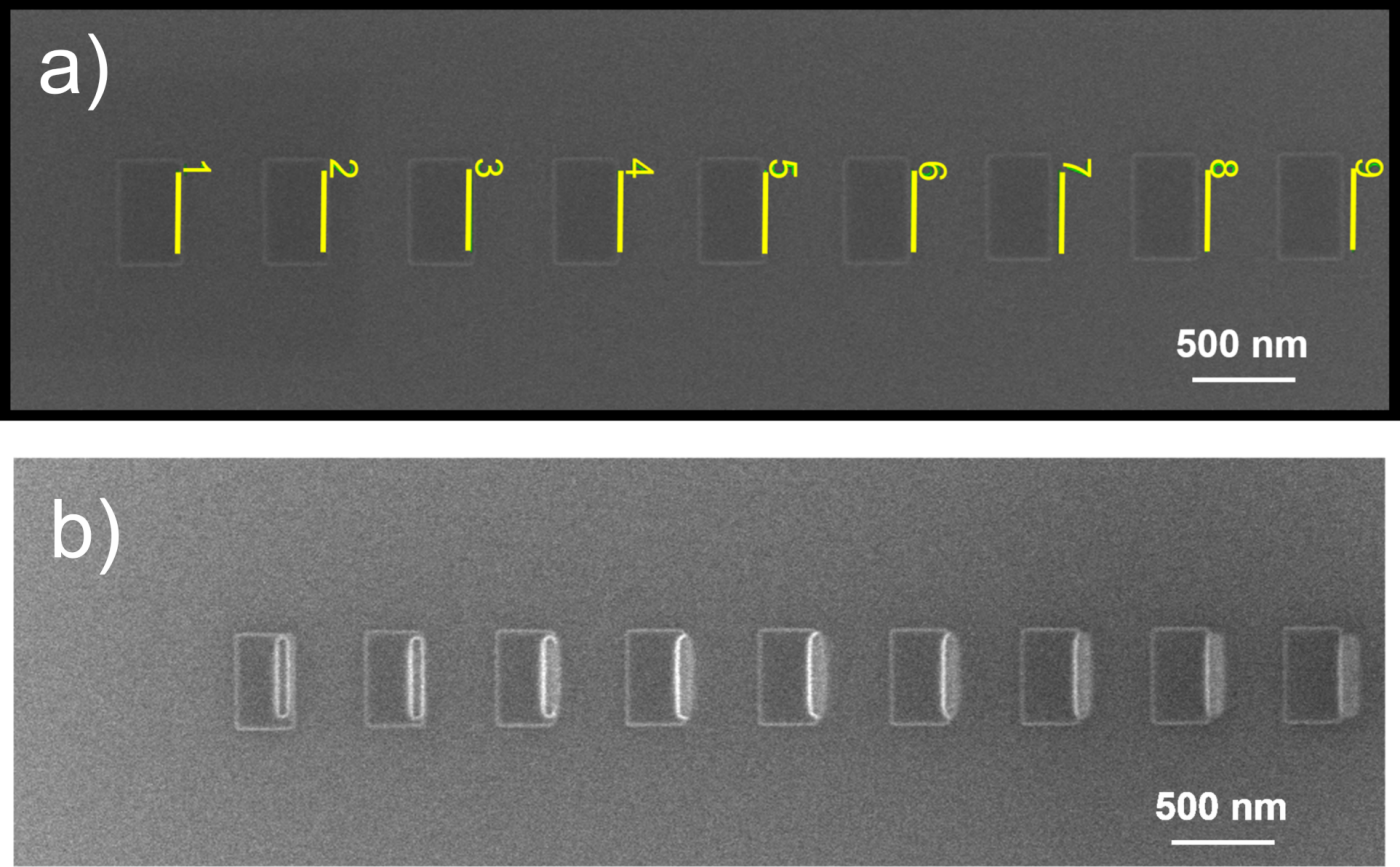
(a) Top view SE image of the array of EBID deposits along with the etching scheme: The array of etch patterns was aligned with the deposit array at increasing distance from the deposit centre from left to right. The nine deposits shown are to be etched and their profile compared with that of the last deposit (not shown) on the far right. (b) Top view SE image of the deposit array after etching.

Ten carbon deposits were made, 300 nm in width and 500 nm in length, identically patterned at a centre-to-centre separation of 700 nm. The height of the deposits was approximately 40 nm. An etch pattern comprising nine lines of 400 nm length was defined to etch the right sidewall of each deposit. This array was aligned such that each subsequent etch was positioned 20 nm farther away from the centre of the corresponding deposit than the previous etch. Figure 3a shows the scheme that was used, beginning with etch 1 (left) that was placed (by inspection) somewhere on the right-hand side of the plane top of deposit 1, to etch 9 (right) located farthest from the centre. Deposit 10, not shown in the figure, was not etched and functioned as the reference. The lines were etched serially from left to right. The distance between the deposits was chosen to be larger than the range of the BSE electrons so that each deposit could be etched independently. Figure 3b shows a quick-scan top view SE image of the array after etching, revealing successful removal of material, indicated by the observed brightening (see Supporting Information File 1, section S2).

**Figure 4:**
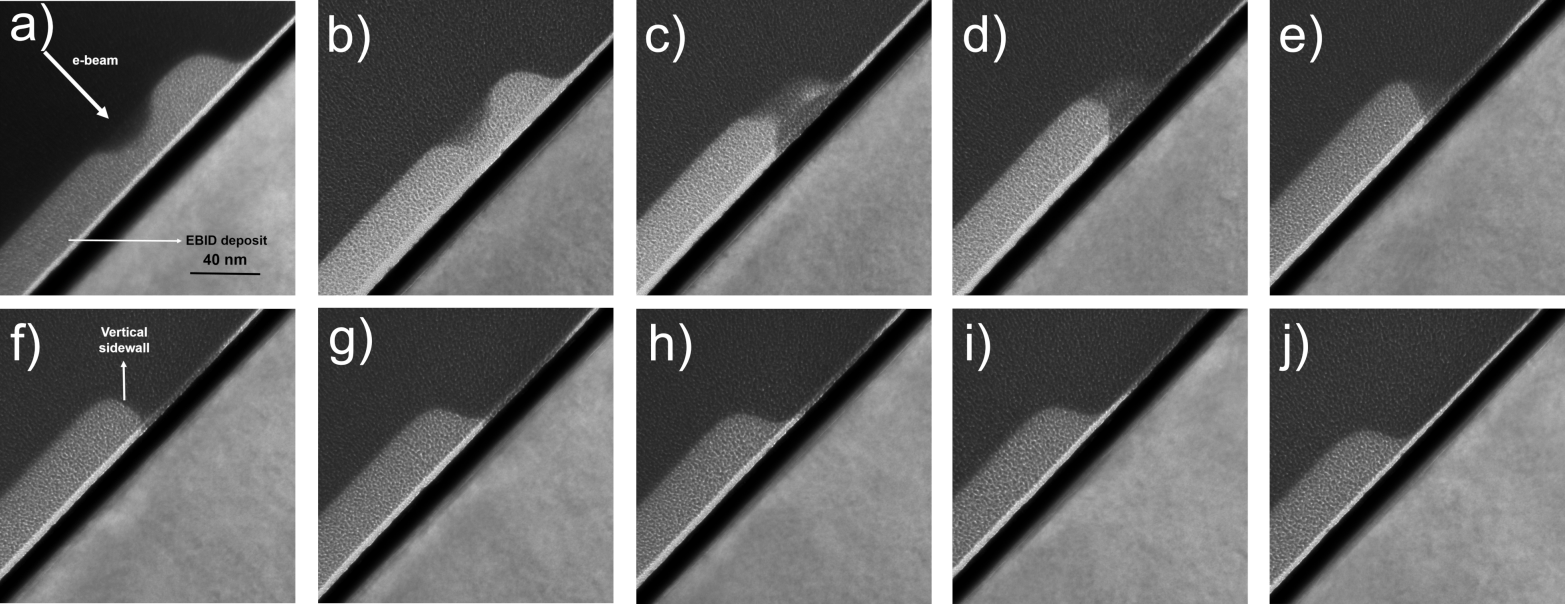
(a–j) Sidewall evolution as a result of the etching series. The frames (a) to (j) show the ten deposits from left to right after etching. The images were acquired in bright-field mode in an FEG Tecnai 20 D239 S-Twin TEM using an acceleration voltage of 200 keV and spot 3. The EBID deposit is indicated in (a), and the white arrow shows the approximate position of the electron beam for EBIE. The successful creation of a vertical sidewall is visible in (f).

A lamella was cut out of this sample as a whole, spanning the entire region from the left of deposit 1 to the right of deposit 10, and imaged in bright-field mode in a Thermo Fisher Scientific FEG Tecnai 20 D239 S-Twin TEM using an acceleration voltage of 200 keV and spot 3. The evolution of the right sidewall is shown in Figure 4, where a clear, albeit surprising, trend is visible. The profile of the as-deposited structure (deposit 10) is shown in Figure 4j for reference. Clearly, etching with the same PE dose at different positions on the slope, separated by as little as 20 nm, results in very different profiles. Although the profiles of etch 1 and etch 2 appear Gaussian as expected from the etching of a plane surface, proceeding outwards brings about the abrupt onset of under-etching. Deposit 3 shows a modified sidewall with the lower half sloping inward, forming a sideways cap, and some material protrudes from the right of the deposit indicating an incomplete etch. Moving further right and, therefore, etching thinner material, etch 4 results in a smoother profile, still capped. Here, the dose was sufficient to perform a complete etch, and the connecting material previously seen adjacent to the deposit is gone. The profile becomes still smoother after etch 5, showing a sidewall that slopes inward completely. 20 nm further to the right, however, the trend seems to reverse, and etch 6 results in the much desired vertical sidewall. This is, therefore, the position where the used etching dose is optimal; upon moving further outward, the sloping sidewall is visible once again with some clipping of the long tail. And finally, on etching sufficiently far away from the centre, the profile remains as deposited. The angular dependence of the SE yield of the deposit alone cannot be responsible for this evolution. According to the simulation, an under-etch or even a perfectly vertical sidewall is impossible to achieve, the best case being a nearly vertical, but still outward-sloping profile. The experimental results therefore demonstrate the need for a new model.

### Modelling of sidewall modification by FEBIE

A realistic model of FEBIE involves precise knowledge of the distribution of electrons generated by the interaction of the primary beam with the substrate and the deposit. In addition, the dissociation of the precursor molecules needs to be modelled, secondary reactions of the etch products need to be taken into account, also the residence time of the fragments on the deposit, the sticking and diffusion of the water molecules, and so on. Although this is a very challenging task, some models were developed [21–22], starting from the continuum model for FEBID [23]. But the etching process unfortunately is not quite as straightforward as the deposition process. For instance, the etching rates are difficult to model when the rate limiting factor is determined by the residence time of the etching products on the surface [22]. As none of these models attempted to describe the evolution of the deposit shape subject to etching, the simple model described above will be slightly extended by including under-etching of the deposit once the etch pit has reached the substrate. As before, the extended model is two-dimensional and the evolution of a deposit geometry is studied as a function of etching location and exposure dose. All units in the model are arbitrary.

The geometry of the deposit is as in Figure 2a, with a flat top and Gaussian sidewalls. The water molecules, the FEBIE precursor, adsorb onto the surface and are assumed to immediately form at least a monolayer. They can be dissociated by the distribution of SE emitted around the point of impact of the primary beam, causing material to be etched. As before the etching profile on a flat deposit is taken as a Gaussian function *G*_d_(*x*)


[1] $$G_{\text{d}}\left(x\right)=S_{\text{d}}e^{\frac{-{\left(x-x_{0}\right)}^{2}}{2\sigma_{\text{d}}^{2}}}$$

where the subscript d indicates the deposit, *x* is the horizontal coordinate, *x*_0_ is the location of the primary beam, *S*_d_ is the etching strength, and σ_d_ is the standard deviation of the function. On a sloped sidewall the etching is enhanced by the factor 1/cos α(*x*), as before. The etching dose is delivered by repeated exposures, the number of which is user defined. As soon as the etching process from the top has reached the (inert) substrate at the primary beam position, the SE generated in the substrate may cause further etching from below. This process starts as a lateral under-etch at the foot of the deposit where adsorbed water molecules get dissociated by the substrate SE. In the simulation, the extent over which the under-etch occurs is limited to a certain length *w* per exposure. The amount of material removed from the bottom of the deposit is assumed to be proportional to the SE distribution of the substrate, also taken as a Gaussian function *G*_s_(*x*)


**Figure 5:**
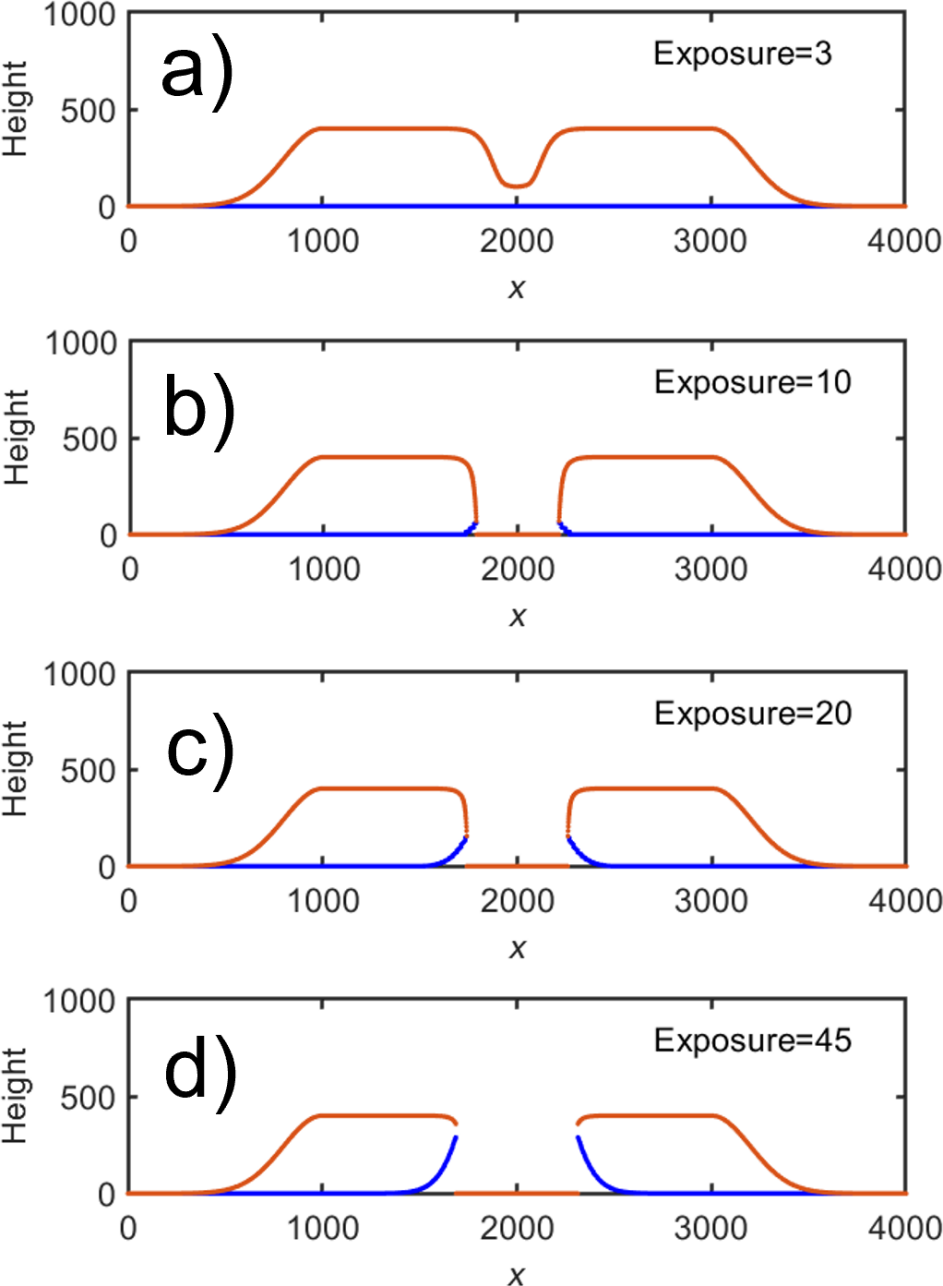
Resulting profiles for continued etching from below for 3 (a), 10 (b), 20 (c), and 45 (d) consecutive exposures, demonstrating the increase in under-etching with dose. The beam was positioned at *x*_0_ = 2000, and the other parameters were σ_d_ = 100, S_d_ = 100, σ_s_ = 180, *S*_s_ = 40, and *w* = 20. The top part of the deposit is drawn in red and the bottom part in blue. All dimensions are in arbitrary units.

[2] $$G_{\text{s}}\left(x\right)=S_{\text{s}}e^{\frac{-{\left(x-x_{0}\right)}^{2}}{2\sigma_{\text{s}}^{2}}}$$

where the subscript s denotes the substrate. With each following exposure, the deposit gets etched further from the top, as long as *G*_d_(*x*) still has some overlap with the deposit. At the substrate, the etch further extends into the deposit over another length *w*, removing material from underneath governed by *G*_s_(*x*). This process is illustrated in Figure 5 for four different exposures of a geometry similar to that in Figure 2a. In Figure 5b it is seen that almost vertical sidewalls are achieved. However, when the dose is increased further, considerable under-etching occurs. A crucial condition for achieving under-etching in this model is that σ_s_ > σ_d_. The relative etching strength is of less importance. Such a situation may well occur in the experiment shown in Figure 3, as at 20 keV primary energy, the radius within which SE2 are created is larger in the Si substrate than in the C deposit (2.7 and 1.6 μm, respectively [24]). Note that the thin Au–Pd and Ti coating is ignored here, as most of the interaction volume will be in the underlying Si substrate at 20 keV. Therefore, the width of the distribution of SE originating from the substrate will be larger than that of the carbon deposit.

**Figure 6:**
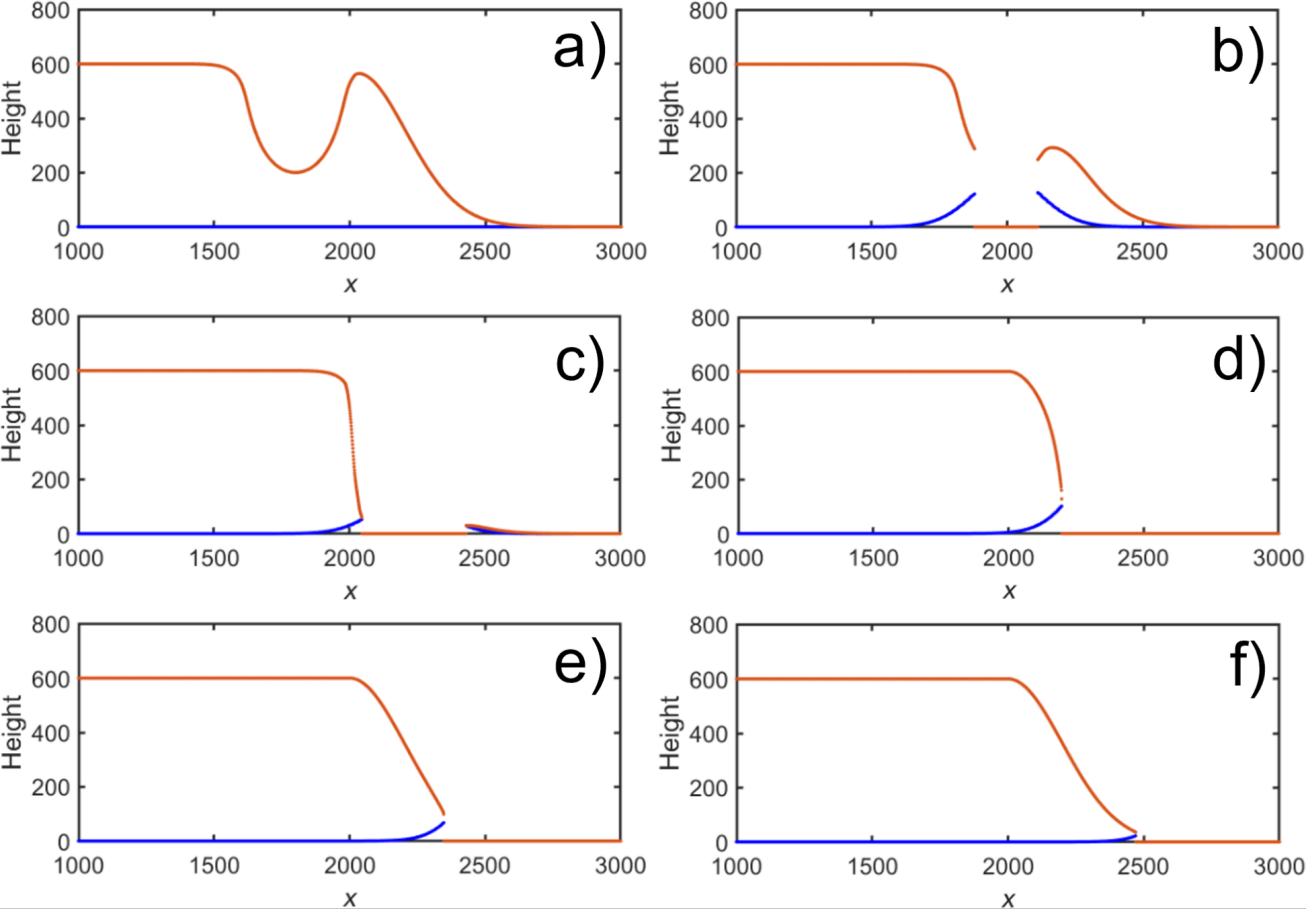
Simulations showing the evolution of the half-Gaussian-shaped sidewall (with σ = 200) of a deposit of height 600 as a function of the etch position. The red lines indicate the top part, as etched from above, and the blue lines the bottom part, as etched from below. In (a–f), the beam was positioned from *x*_0_ = 1800 to *x*_0_ = 2800 in steps of 200. All other parameters were kept constant at σ_d_ = 100, *S*_d_ = 5, σ_s_ = 140, *S*_s_ = 5, and *w* = 20. The number of exposures was 80. All dimensions are in arbitrary units.

To simulate the experimental results shown in Figure 4, etching a series of positions on the Gaussian-shaped sidewall of a flat deposit was simulated. The number of PE exposures was 80 and kept the same at all locations. The parameters of the Gaussian functions were σ_d_ = 100, *S*_d_ = 5, σ_s_ = 140, and *S*_s_ = 5. The distance over which the under-etching occurs per exposure was *w* = 20. The evolution of the profile is shown in Figure 6a–f. The primary beam was moved from *x* = 1800 (Figure 6a) to *x* = 2800 (Figure 6f) in steps of 200.

It is at once evident that the trend of the experiment is reproduced qualitatively. On moving to the right, as soon as sufficient material has been removed for the substrate to be exposed to the beam, additional etching by SE from the substrate takes place giving rise to the under-etch visible in Figure 6b. (Note that the blue and the red lines do not connect because of the digitization of the simulation. The profile is vertical at the locations where they should connect). It is important to note that this effect is essential in producing an under-etch, and etching from the top alone would always result in an outward slope. Further, the extent of under-etch depends on the ratio between the SE distribution widths of the deposit and the substrate, which has been chosen arbitrarily here to simply demonstrate the phenomenon. As in Figure 4c, there is a small amount of material remaining to the right of the deposit in Figure 6b, moving further to the right, which has decreased in Figure 6c where an almost vertical sidewall is achieved. On moving further away, Figure 6d and Figure 6e, the slope is less affected with solely the long tail etched away. Finally, in Figure 6f, the profile is almost unchanged with respect to the as-deposited structure.

## Discussion

Although the trends in Figure 4 and Figure 6 are qualitatively similar, several differences are visible too. The under-etch remains quite pronounced in the simulation when moving down the slope, much more than in the experimental results. This could be due to the fact that a simple analytical model is not sufficient to fully simulate the experimental conditions. Further, the parameters used in the model are in arbitrary units. They were chosen such that the experimentally observed trend could be replicated, and not from physical considerations. The parameters related to etching, in particular, and their values relative to the deposit geometry might therefore be unrealistic. This is supported by the fact that attempts to scale the parameters against the experimentally observed values were unsuccessful. One of the main issues is that the regime in which the etching takes place is unknown. It was noticed that a small change in the pressure of water vapour led to a significant change in the etching rate, suggesting that the process is gas-limited (see Supporting Information File 1, section S3). The role of diffusion could therefore be significant. The diffusion rate of adsorbed contamination is known to be enhanced by the presence of water layers [21]. But since the relevant quantities are hard to measure, the diffusion rate has not been included in the model, nor have some other factors such as scattering, porosity, and secondary etch product reactions.

Another major difference is that in the experiment, a line is being etched with a PE beam having a finite spot size. The effect of pixel dwell time, pixel overlap and multiple passes has not been taken into account, all of which could play an important role in circumstances involving surface diffusion. The goal of this model is only to provide a qualitative explanation of sidewall evolution under etching, which is not addressed by existing models and which is necessary for carrying out a well-controlled experiment.

In the experiment, the positioning of the etch series with respect to the deposit array was performed by eye using the same field of view (8 μm) each time. As this is rather large, it is reasonable to assume that when the experiment was repeated for confirmation, even assuming that the deposits were identical, etching might have taken place at a somewhat different location in each instance. The images showed that the trend in the profile is reproduced, although a certain profile may occur at a different location each time the series is repeated. This means that, at least within a range of 100 nm on the sidewall, given a position, a suitable electron dose would result in a vertical sidewall. This suggests that etching can be carried out at any position on the sidewall if the right dose can be applied to make it vertical. From a practical point of view, it would be advantageous if this entire process, etching as well as imaging, could be implemented in situ in the SEM. The above result is encouraging because it suggests that if the sidewall evolution could somehow be monitored, one could begin etching at an arbitrary position (within a certain range, still determined by eye) and stop when the desired profile is attained.

**Figure 7:**
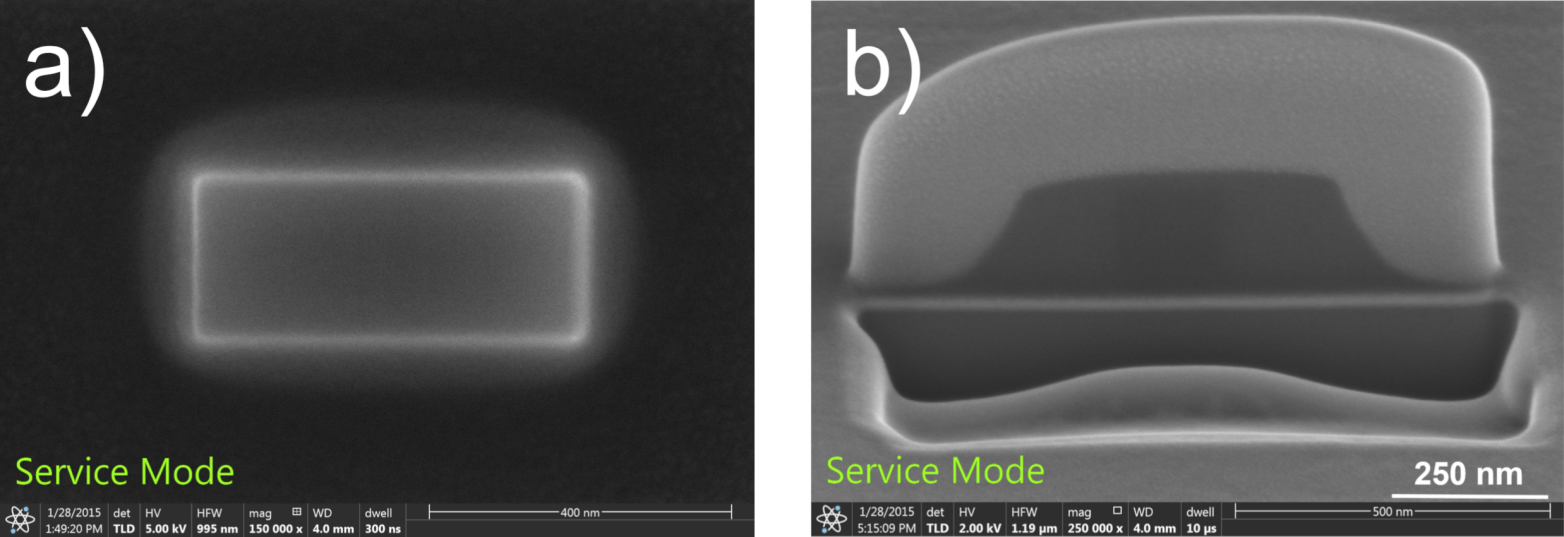
(a) Top view SE image and (b) FIB cross section of an as-deposited FEBID structure.

To demonstrate this, in the following experiment, SE imaging is used to monitor the etching. As the SE emission from the sidewall is known to depend on the angle between the beam and the surface, an increase in SE emission is expected as the sidewall angle approaches 90°. For a sufficiently thick deposit, this change could lead to edge highlighting in the SE image. A 200 nm thick carbon FEBID deposit was fabricated, which would be thick enough to image with SE as well as using FIB cross sectioning. The top view SE image and cross section of the reference structure are shown in Figure 7. Such a deposit was then exposed to FEBIE at both sidewalls. A beam energy of 5 keV was used to speed up the process since the deposit here is significantly thicker. As the exact position for carrying out the etching could not be determined in advance, an area approximately 60 nm wide and 600 nm long was exposed to a 5 keV electron beam and 1.6 nA current in the presence of water.

**Figure 8:**
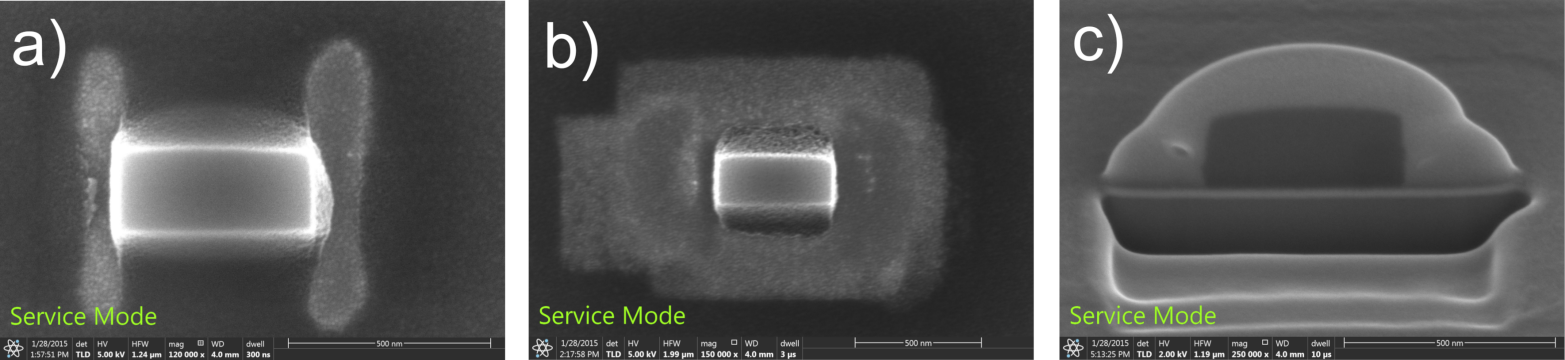
(a) Top view SE image after etching of the sidewall and (b) after the cleaning step. (c) FIB cross section of deposit after sidewall etching clearly demonstrating the creation of vertical sidewalls.

This pattern was first positioned at an arbitrary distance from the sidewalls, and the etching was carried out until a brightening of the area was observed (Figure 8a), signifying the removal of carbon ([25], and Supporting Information File 1, section S2). The patterns on both sides were then moved closer to the deposit, and the process was repeated until a sharp highlighting of the edges was observed, suggesting the formation of nearly vertical walls. A cleaning step was then performed, using a large area etch (as in [25]), to remove carbon from all around the deposit up to a few hundred nanometres. The SE image of the resultant structure is shown in Figure 8b. The deposit was then covered with a protective layer of Pt/C as before, and a cross section was made using a FIB (Figure 8c), clearly demonstrating the creation of vertical sidewalls.

## Conclusion

A new technique combining FEBID and FEBIE has been developed to fabricate structures with vertical sidewalls. The Gaussian profile of as-deposited carbon FEBID structures has been modified by etching with water, and controlled tuning of the sidewall angle has been demonstrated, including the creation of vertical sidewalls. By simply varying the etch position on the sidewall using the top view SE image for reference, the slope of the deposit can be tuned from negative (outward) to positive (inward). The evolution has been studied in detail by high-resolution imaging in a TEM.

A surprising trend not indicated by the simple model based on etching due to SE from the deposit, which was the starting point of the study, was observed. While etching proceeds as expected for very low doses, under-etching was observed at higher doses. An analytical model was developed that nevertheless incorporates the effect of water adsorption in a simple manner. In this model too, the etching is governed by SE, but once the deposit material at a location has been removed, exposing the bare substrate, water molecules are adsorbed there. The SE generated from the substrate by continued exposure to the beam are now effective in removing the deposit material from below, resulting in under-etching. The role of the substrate, which is assumed not to be altered by the etching process, is crucial; without it, etching would always result in an outward sloping sidewall, whose angle varies with position and dose.

The sidewall etching experiment has also been carried out in situ in an SEM. Making use of the phenomenon of enhanced SE emission from an edge, the evolution of the sidewall angle during etching was continuously monitored using the SE signal. It has been demonstrated that this technique is sufficiently sensitive to determine the dose at which the sidewall angle becomes 90°.

The method described here to make vertical sidewalls of FEBID deposits has the potential to make FEBIP a more competitive technology for lithography applications.

## Experimental

The FEBID and FEBIE experiments were carried out in a Thermo Fisher Scientific Helios 650 Dual beam system equipped with two gas injection systems (GISs) for precursor delivery. The GIS nozzles were adjusted to be 150 μm above the sample and at a distance of 100 μm from the centre of the field of view. The precursors chosen were the same as in an earlier study to remove carbon interconnects using FEBIE with water [25], namely dodecane (C_12_H_26_) for the deposition of carbon and crystals of MgSO_4_·7H_2_O for etching with water. Both precursors were let into the chamber at room temperature. The base pressure in the specimen chamber was between 2 × 10^−6^ and 4 × 10^−6^ mbar. During patterning (both FEBID and FEBIE) the pressure was in the range of 2.5 × 10^−5^ and 4.5 × 10^−5^ mbar. Following a few hours of deposition, the chamber would take increasingly longer to pump down, and electron beam-induced sample contamination was observed to increase. This was likely due to dodecane sticking to the walls of the chamber and other open surfaces. Additionally, on letting in water after deposition, the contamination level was found to be higher. This is consistent with reports of increased diffusion of hydrocarbons in the presence of adsorbed water layers. Therefore, to maintain clean working conditions, all carbon depositions were performed in succession, after which the chamber was allowed to pump down for at least 2 h, and overnight when possible. The etching experiments were then performed in succession.

The carbon deposits for the TEM inspection were patterned with a 20 keV beam with 3.2 nA of current, a pitch between exposure points of 5 nm, and a dwell time of 1 μs; the pattern was repeated for 3000 passes. The etching parameters were 20 keV, 3.2 nA, a pitch of 1 nm, a dwell time of 10 μs, and 35000 passes. It should be noted that for the etching process the electron beam current and precursor flux were carefully selected, as they were found to influence the process significantly (see Supporting Information File 1, sections S2 and S3).

The cross-sectional profiles were obtained by FIB milling. Deposits were first covered with a protective Pt/C cover, at least 1.5 μm thick, by FEBID from MeCpPtMe_3_. Then the sample was tilted by 52° and milled with a gallium FIB. The cross-sectional profile was imaged at low energy (2 keV) with the SE detector.

## Supporting Information

The Supporting Information contains three sections. Section S1 provides more detailed information on the sidewall modification simulation, section S2 addresses the influence of the electron current on FEBIE, and section S3 discusses the influence of the gas flux on FEBIE.

File 1 Additional experimental data.

## Data Availability

The data that supports the findings of this study is available from the corresponding author upon reasonable request.
