# Supplementary material for: Sidewall angle tuning in focused electron beam-induced processing
Source: Beilstein J Nanotechnol. 2024 Apr 23;15:447–56. doi: 10.3762/bjnano.15.40 (PMC11070960; doi:10.3762/bjnano.15.40)
Supplement: File 1 — Additional experimental data. [file Beilstein_J_Nanotechnol-15-447-s001.pdf]

BEILSTEIN JOURNAL OF NANOTECHNOLOGY

Supporting Information

for

Sidewall angle tuning in focused electron beam-induced processing

Sangeetha Hari, Willem F. van Dorp, Johannes J. L. Mulders, Piet H. F. Trompenaars, Pieter Kruit and Cornelis W. Hagen

Beilstein J. Nanotechnol. **2024,** *15,* 447–456. doi:10.3762/bjnano.15.40

Additional experimental data

License and Terms: This is a supporting information file under the terms of the Creative Commons Attribution License (https://creativecommons.org/licenses/by/4.0). Please note that the reuse, redistribution and reproduction in particular requires that the author(s) and source are credited and that individual graphics may be subject to special legal provisions.
The license is subject to the *Beilstein Journal of Nanotechnology* terms and conditions: (https://www.beilstein-journals.org/bjnano/terms)

S1 Sidewall slope modification – proof of principle simulation

A large EBID deposit was simulated on a substrate to have a flat top and symmetric sidewalls defined by half-Gaussian functions as shown in Figure S1.1.

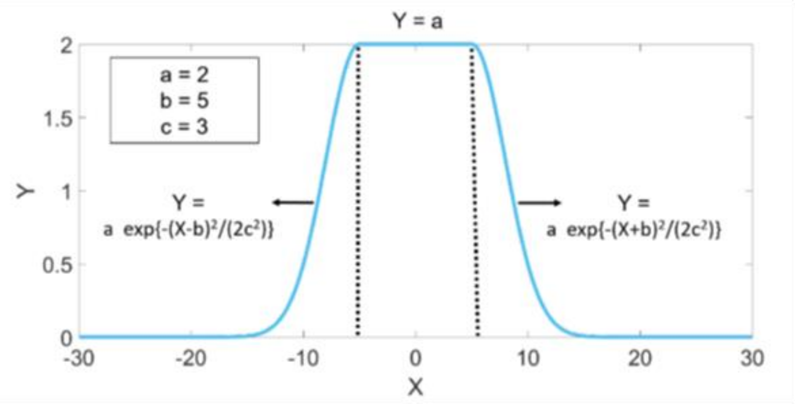

Figure S1.1: Simulated FEBID deposit with a flat top (Y=a). The sidewalls are defined by half-Gaussian functions as indicated in the figure. The values used here are a=2, b=5, c=3.

The etch profile was defined similarly by a Gaussian function: $Y_2 = a_2 e^{\frac{-(X-b_2)^2}{2c_2^2}}$. The results of etching the deposit are shown in Figure 2 of the paper where the red curve in (a) and (b) represents the etch profile on a plane surface. When the beam is positioned on the flat top of the deposit for etching (a), the material removed results in a Gaussian profile as shown by the green curve. The situation is different when etching takes place on a sloping surface. The SE yield from the slope is enhanced by a factor proportional to 1/cosα where α is the angle between the beam and the normal to the surface at the point of incidence. The proportionality constant "k" now determines the strength of etching. The dimensions of the deposit and the strength of etching are variables in the model and for the purpose of this simulation, all parameters are in arbitrary units. Upon positioning the beam symmetrically, but at an arbitrary distance from the centre, on each of the sloping sidewalls, the deposit is etched by SE's. The

etching was performed in 7 steps with a fixed beam position and etching strength and the resultant profile (Y_3) of the FEBID deposit evolves as:

$$Y_3(i) = Y_3(i-1) - \frac{kY_2}{[cos\alpha]_{at\ Y_3(i-1)}}$$

where ”i” represents the etching step. The slope of the sidewall (Y_3) evaluated for the step ”(i-1)” is used to determine the enhancement in SE yield as shown by the second term in the equation. Convolved with the etch profile Y_2 together with the proportionality constant ”k”, this represents the material removed from the current profile Y_3(i-1) to give the resultant deposit profile Y_3(i). The sidewall slope is negative (i.e., sloping outwards) at the beginning as shown by Figure 2. After the first etching step, this slope becomes more negative (moving inward) indicated by the profile marked ”Step 1” in Figure 2b. The process continues with the slope becoming increasingly more negative with every etching step until a well defined deposit with nearly vertical sidewalls is achieved in ”Step 7”. It should be pointed out here that the etching strength in (a) was chosen to be low to give an indication of the initial stages of etching. For a higher value such as that in (b), the etching would result in removal of material all the way to the substrate. This would be unrealistic because after the initial stages of etching, the deposit surface is no longer plane and the enhanced SE emission from a sloping surface must be taken into account similarly to what was described in (b). This simple model shows that FEBIE can be applied to as-deposited FEBID structures to achieve vertical sidewalls.

S2 Influence of electron current in FEBIE.

During FEBIE, simultaneous deposition of carbon can take place by FEBID from contamination present in the specimen chamber. By altering the electron flux, switching between the two processes has been demonstrated [1]. Exposing a shallow

carbon deposit such as a contaminated gold-palladium surface to a line scan with low current (100 pA) in the presence of water vapour resulted in no etching (cleaning) over the dose range (number of passes ranging from 300 to 1000). Instead, a deposit was observed in the SE image (Figure S2.1a)). This is due to insufficient electron-water collisions for etching. The electron dose was however enough to deposit carbon from the chamber contamination which led to the darkening seen. Continued electron exposure would then result in more carbon deposition, leading to a broadening of the line as in Figure S2.1b but no etching. Exposing such a deposit to high current (1.6 nA) and 1000 passes, while all other parameters were kept unchanged resulted in a brightening of the scanned line (Figure S2.1c) as expected from this reasoning. Additionally, some carbon deposition is observed on the rim of the etched line, which can also be explained as coming about due to the tails of the electron beam profile. A higher dose (5000 passes) at the same current resulted in a broadening of the etched/cleaned line (Figure S2.1d) along with the deposition of carbon on the outside as before, lending experimental support to this explanation.

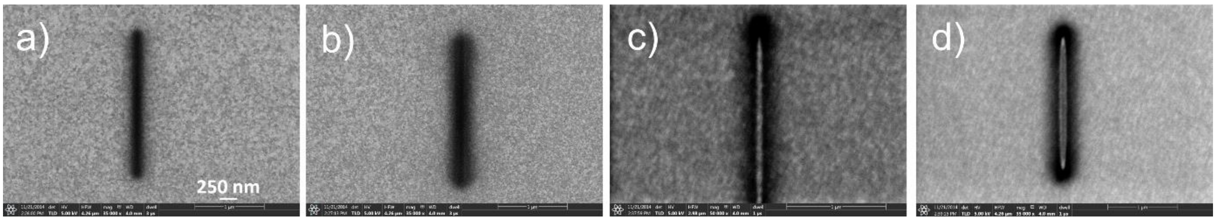

Figure S2.1: The influence of electron current on the etching of a contaminated gold-palladium surface. a), b) FEBIE of a line pattern at low current (5 keV, 100 pA, 300 (a), 1000 (b) passes, dwell time = 500 μs) can be seen to be unsuccessful in cleaning/ etching the surface contamination. Instead, darkening of the exposed line is observed which broadens on increasing the dose in b). c), d) FEBIE at high current (5 keV, 1.6 nA, 1000 (c), 5000 (d) passes) can be seen to cause brightening of the exposed line, indicating etching. The darkening on the outside of the line can be understood in terms of deposition from chamber hydrocarbons caused by the tails of the electron beam.

S3 Influence of gas flux in FEBIE

The use of a high current (a few nA) for etching suggests that the process is not current limited. The gas flux is therefore expected to be a critical parameter. This was verified using the same current to expose lines on two identical carbon deposits when a chamber pressure of 0.9 × 10^{-5} mbar of water vapour resulted in unsuccessful etching (Figure S3.1a) whereas at a pressure of 2.5 × 10^{-5} mbar the advent of etching was visible (Figure S3.1b). The local gas flux incident on the sample estimated in the two cases is 4 × 10^{17} molecules/cm^2/s and 1.6 × 10^{18} molecules/cm^2/s, respectively. This difference was therefore sufficient to bring about a change in the balance between FEBID and FEBIE and gives an idea of the sensitivity of the process to the gas flux. The choice of current and gas pressure, while clearly significant, are not independent of each other and together govern the balance between etching and the deposition of contamination.

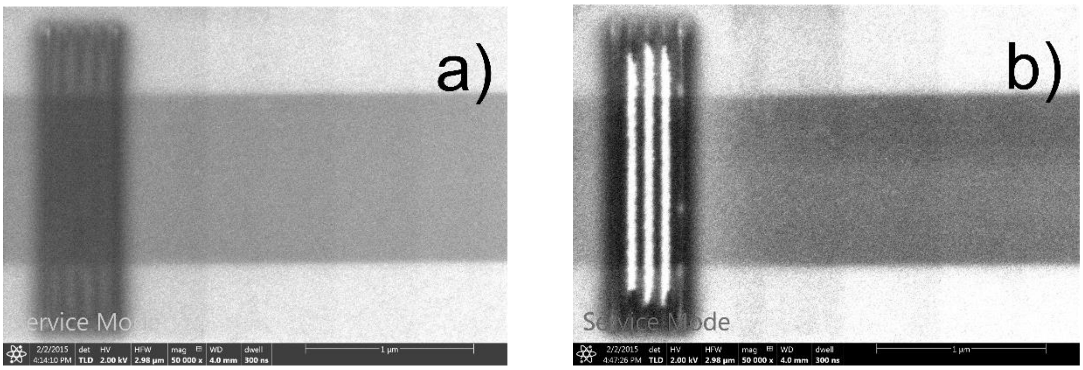

Figure S3.1: FEBIE at gas pressures of 0.9 × 10^{-5} mbar (a) and 2.5 × 10^{-5} mbar (b). The local gas flux incident on the sample in the two cases is 4 × 10^{17} molecules/cm^2/s and 1.6 × 10^{18} molecules/cm^2/s, respectively.

Funding

This work is supported by NanoNextNL, a micro and nanotechnology programme of the Dutch Government and 130 partners.

References

1. Toth, M.; Lobo, C.J.; Hartigan, G.; Ralph Knowles, W. *J. Appl. Phys.* **2007**, *101*, 054309. doi:10.1063/1.2437667
